# Cardiovascular Risk Following Conversion to Belatacept From a Calcineurin Inhibitor in Kidney Transplant Recipients: A Randomized Clinical Trial

**DOI:** 10.1016/j.xkme.2022.100574

**Published:** 2022-11-18

**Authors:** Obbo W. Bredewold, Joe Chan, My Svensson, Annette Bruchfeld, Johan W. de Fijter, Hans Furuland, Josep M. Grinyo, Anders Hartmann, Hallvard Holdaas, Olof Hellberg, Alan Jardine, Lars Mjörnstedt, Karin Skov, Knut T. Smerud, Inga Soveri, Søren S. Sørensen, Anton-Jan van Zonneveld, Bengt Fellström

**Affiliations:** 1 Department of Nephrology, Leiden University Medical Center, Leiden, The Netherlands; 2 Department of Renal Medicine, Akershus University Hospital, Lørenskog, Norway; 3 Department of Clinical Medicine, Aalborg University Hospital, Aalborg, Denmark; 4 Department of Health, Medicine and Caring Sciences, Linköping University, Linköping, Sweden; 5 Department of Renal Medicine, Karolinska University Hospital and CLINTEC Karolinska Institutet, Stockholm, Sweden; 6 Department of Medical Science, Renal Unit, University Hospital, Uppsala, Sweden; 7 Department of Clinical Sciences, Faculty of Medicine and Health Sciences, University of Barcelona, Barcelona, Spain; 8 Department of Transplantation Medicine, Oslo University Hospital, Oslo, Norway; 9 Department of Internal Medicine, School of Medical Sciences, Örebro University, Örebro, Sweden; 10 Department of Cardiovascular and Medical Sciences, University of Glasgow, Glasgow, UK; 11 Division of Transplantation, Department of Surgery, Sahlgrenska University Hospital, Göteborg, Sweden; 12 Department of Renal Medicine, Aarhus University Hospital, Denmark; 13 Smerud Medical Research International AS, Oslo, Norway; 14 Department of Nephrology, Copenhagen University Hospital, Copenhagen, Denmark

**Keywords:** Kidney transplantation, randomized clinical trial, belatacept, calcineurin inhibitors, tacrolimus, cardiovascular disease, mortality, pulse wave velocity, blood pressure, arterial stiffness

## Abstract

**Rationale & Objective:**

In kidney transplant recipients (KTRs), a belatacept-based immunosuppressive regimen is associated with beneficial effects on cardiovascular (CV) risk factors compared with calcineurin inhibitor (CNI)–based regimens. Our objective was to compare the calculated CV risk between belatacept and CNI (predominantly tacrolimus) treatments using a validated model developed for KTRs.

**Study Design:**

Prospective, randomized, open-label, parallel-group, investigator-initiated, international multicenter trial.

**Setting & Participants:**

KTRs aged 18-80 years with a stable graft function (estimated glomerular filtration rate > 20 mL/min/1.73 m^2^), 3-60 months after transplantation, treated with tacrolimus or cyclosporine A, were eligible for inclusion.

**Intervention:**

Continuation with a CNI-based regimen or switch to belatacept for 12 months.

**Outcomes:**

Comparison of the change in the estimated 7-year risk of major adverse CV events and all-cause mortality, changes in traditional markers of CV health, as well as measures of arterial stiffness.

**Results:**

Among the 105 KTRs randomized, we found no differences between the treatment groups in the predicted risk for major adverse CV events or mortality. Diastolic blood pressure, measured both centrally by using a SphygmoCor device and peripherally, was lower after the belatacept treatment than after the CNI treatment. The mean changes in traditional cardiovascular (CV) risk factors, including kidney transplant function, were otherwise similar in both the treatment groups. The belatacept group had 4 acute rejection episodes; 2 were severe rejections, of which 1 led to graft loss.

**Limitations:**

The heterogeneous baseline estimated glomerular filtration rate and time from transplantation to trial enrollment in the participants. A limited study duration of 1 year.

**Conclusions:**

We found no effects on the calculated CV risk by switching to the belatacept treatment. Participants in the belatacept group had not only lower central and peripheral diastolic blood pressure but also a higher rejection rate.

**Funding:**

The trial has received a financial grant from 10.13039/100002491Bristol-Myers Squibb.

**Trial Registration:**

EudraCT no. 2013-001178-20.

## Visual Abstract

**Figure:**
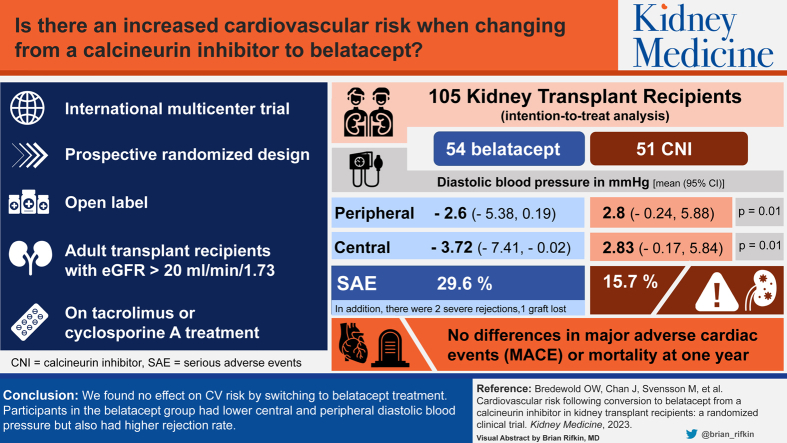

**Plain-Language Summary**

In 2013-2014, belatacept was believed to be superior to cyclosporin A in terms of its CV effects after kidney transplantation; however, not many studies had compared belatacept with tacrolimus. The use of a CV risk calculator instead of hard endpoints provided the chance to investigate the 2 regimens after a relatively short follow-up. The difference in the CV risk was smaller than we expected, although belatacept did lower the blood pressure. Our study again showed that an early prescription of belatacept carries the risk of rejection. Our main learning point is that belatacept, after 15 years of use, should still be regarded as a treatment option for individuals who suffer from tacrolimus-related side effects; however, it is unsuitable for broad implementation for all kidney transplant patients, despite its promising introduction.

The risk of cardiovascular disease (CVD) in patients with kidney failure is much higher than in the general population across all age groups.^1^^,^^2^ Although a successful transplant reduces this risk significantly, kidney transplant recipients (KTRs) still have an annual cardiovascular (CV) event rate of 3.5%-5%.^3^ Accordingly, CVD remains one of the leading causes of death in KTRs.^4^^,^^5^ Managing a transplanted patient should therefore include CV risk reduction measures to improve both graft and patient outcomes. The current guidelines for the prevention of CVD are based on the data from the general population and from studies specifically targeting CVD in KTRs.^6^ In addition to addressing the traditional risk factors for CVD, such as lifestyle choices, hypertension, hyperlipidemia, and diabetes, KTRs present the following 2 potentially modifiable factors: kidney graft function and type of immunosuppressive maintenance regimen.

First, evidence indicates that a declining graft function and graft loss are the potentially modifiable risk factors for CVD and all-cause mortality in this population, which make the strategies for optimizing the graft function important.^7^^,^^8^ Second, among the immunosuppressive drugs used for transplantation, both steroids and calcineurin inhibitors (CNIs) are associated with adverse CV side effects.^9^ Therefore, attempts have been made to minimize or eliminate their use. Although these have led to reasonably safe steroid-free regimens,^10^, ^11^, ^12^ CNIs are still the cornerstone of immunosuppression in modern solid organ transplantation. Early graft survival improved greatly after the introduction of cyclosporine A (CsA) in the early 1980s,^13^ and tacrolimus (TAC) has been the CNI of choice since the 1990s.^14^ Despite the benefits of CNIs in the early posttransplant period, they have dose-dependent side effects, including posttransplant diabetes mellitus, hypertension, hypercholesterolemia, and nephrotoxicity, leading to a progressive decline in kidney graft function.^15^, ^16^, ^17^, ^18^, ^19^ Therefore, there is an ongoing incentive for the development of novel immunosuppressive agents without the side effects of CNIs.

Belatacept, a modified form of cytotoxic T lymphocyte–associated antigen-4-Ig , binds to CD80 and CD86 on antigen presenting cells, thus blocking CD28-mediated costimulation of T cells. The Belatacept Evaluation of Nephroprotection and Efficacy as First-line Immunosuppression Trials (BENEFIT) have shown promise for belatacept as an option for designing a more favorable immunosuppressive regimen.^20^, ^21^, ^22^, ^23^, ^24^ In brief, despite the higher rates of early rejection, the relative risk of death or graft loss after 7 years was reduced by 43% in patients treated with belatacept versus CsA-treated patients, and the estimated glomerular filtration rate (eGFR) in the belatacept group was on average 22 mL/min/1.73 m^2^ higher than that in the CsA group. Furthermore, in a metaanalysis comparing belatacept with CNIs, treatment with belatacept was associated with lower blood pressure, a lower incidence of diabetes, and a more favorable lipid profile.^25^

However, it is not yet proven whether these findings translate into overall CVD reduction. Soveri et al^26^ previously developed a risk calculator for CVD and all-cause mortality for use in KTRs. The group later used the data of the BENEFIT and Belatacept Evaluation of Nephroprotection and Efficacy as First-line Immunosuppression Trial-EXTended criteria donors (BENEFIT-EXT) trials to calculate the potential benefit associated with belatacept treatment and found a substantial calculated 7-year risk reduction for major adverse cardiac endpoints (MACE) and mortality by converting from CsA to belatacept.^27^

A shortcoming of belatacept treatment that has hindered its implementation in kidney transplantation has been the relatively high rate of early rejection, as well as the lack of studies comparing its efficacy with low-dose TAC, the current standard of care in KTRs. In the present study, our aim was to investigate the following: (1) the effects of conversion from a low-dose CNI-based therapy to belatacept on the estimated risk of CVD and all-cause mortality by using the aforementioned risk calculator developed by Soveri et al,^26^^,^^27^ validated for use in KTRs and (2) the changes in the traditional markers of CV health, as well as the measures of arterial stiffness.

## Methods

### Study Design

This was a prospective, randomized, open-label, parallel-group, investigator-initiated, and international multicenter trial (EudraCT no. 2013-001178-20). Patients were randomized in a 1:1 ratio to either continue treatment with a CNI-based regimen or switch to belatacept for a study duration of 12 months. An open design was chosen because CNIs were given as tablets daily and belatacept was administered as infusion every 4 weeks.

Patients were recruited from 9 transplant centers in Denmark (DK), the Netherlands (NL), Norway (NO), and Sweden (SE). KTRs aged 18-80 years with a stable graft function (eGFR > 20 mL/min/1.73 m^2^), 3-60 months after transplantation treated with TAC or CsA, were eligible for inclusion. Patients were excluded if they were Epstein-Barr virus IgG seronegative, had severe de novo or recurrent kidney disease, had a history of vascular or antibody-mediated rejection in the present transplant, or had a history of recent malignancy.

The study was approved by the local ethics committees (NL P15.015, NO 2013/2367, SE 2013/362, DK 1-10-72-119-4). Written informed consent was obtained from all patients, and the trial was performed in accordance with the Declaration of Helsinki and the principles of Good Clinical Practice.

### Study Medication

**Figure 1:**
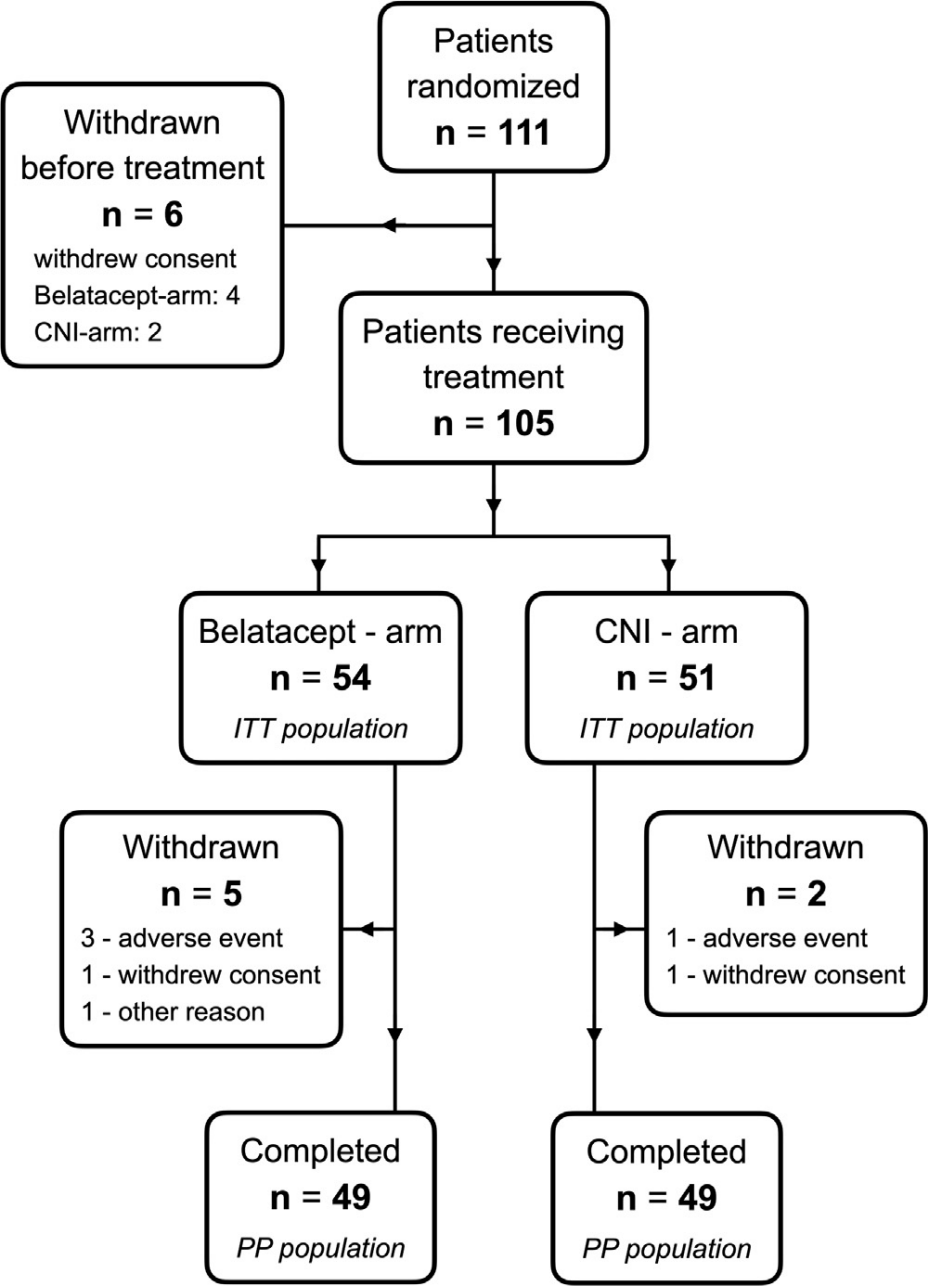
Conversion and dosing scheme. Abbreviations: CNI, calcineurin inhibitor; ITT, intention-to-treat; PP, per protocol.

For patients randomized to the study arm with belatacept, the previous CNI treatment (TAC or CsA) was tapered in the initial period as follows: 100% on day 1, to 70%-80% on day 7, to 40%-60% on day 15, 20%-30% on day 23, and none on day 29 and beyond. Belatacept was dosed 5 mg/kg intravenously on days 1, 15, 29, 43, and 57 and then every month thereafter in the 12-month study period (Fig 1). Patients randomized to the control group with continuation of CNI treatment were to maintain trough levels of CsA between 75 and 200 ng/mL and of TAC between 5 and 10 ng/mL. Both the groups were to continue their underlying immunosuppressive regimen consisting of mycophenolate mofetil (MMF) or mammalian target of rapamycin inhibitor and corticosteroids. Any other concomitant medication necessary to maintain the patients’ baseline condition or to treat a coexisting disease was permitted. Control of blood pressure, glucose, and lipid parameters was left to the treating physicians, according to the local practice.

### Efficacy Assessment and Procedures

The primary endpoint of this trial was the estimated CV risk using a prediction model developed for KTRs by Soveri et al.^26^^,^^27^ The prediction model was constructed on the basis of the Assessment of Lescol in Renal Transplantation extension trial data^28^ and later validated with the Patient Outcomes in Renal Transplantation clinical database.^29^

**Figure 2:**
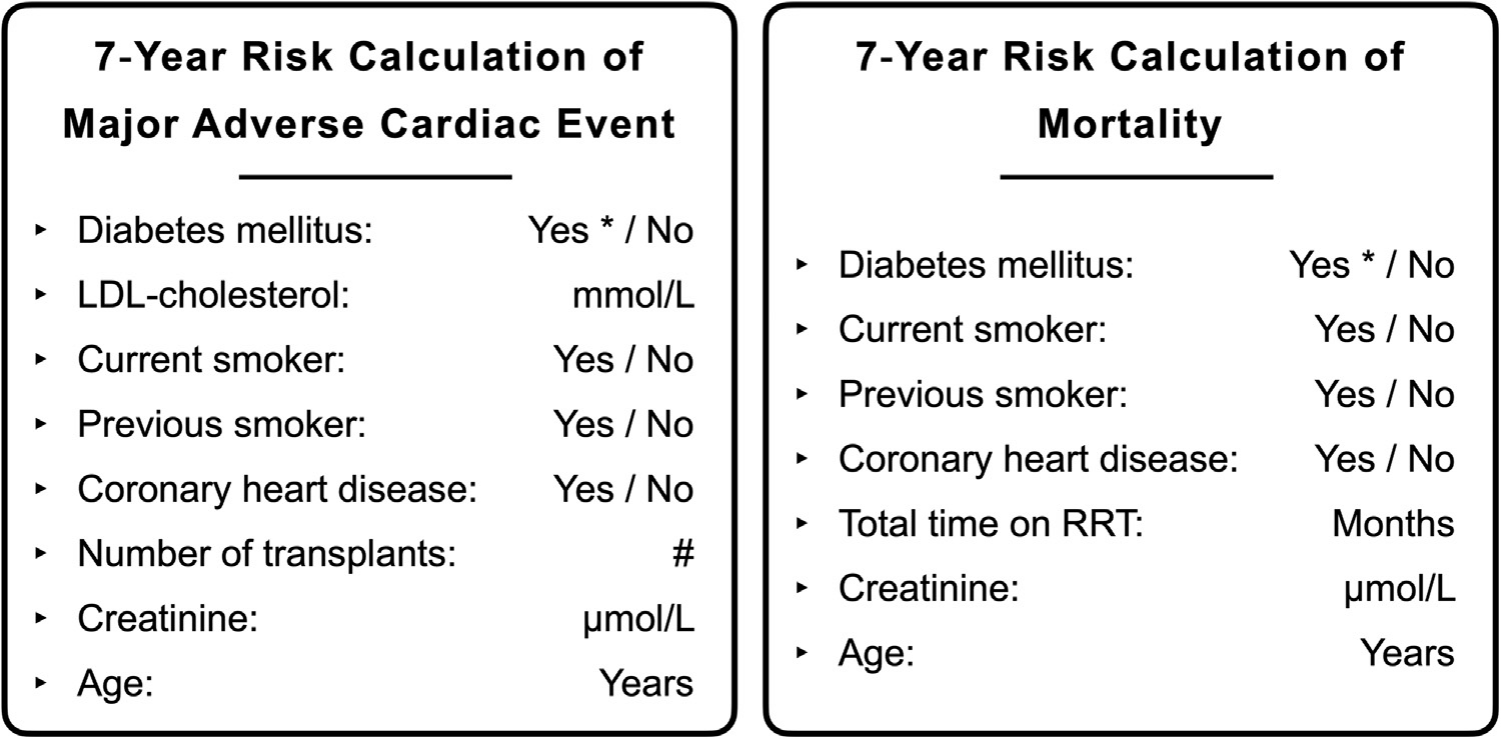
Cardiovascular risk calculator for kidney transplant recipients (Soveri et al^26^, 2012). List of variables used in the cardiovascular risk calculator. ∗Includes posttransplant diabetes mellitus. Abbreviations: LDL, low-density lipoprotein; RRT, renal replacement therapy (including dialysis and transplantation).

The estimated 7-year risk of MACE and all-cause mortality in the 2 treatment groups was calculated as a linear combination of the following variables: age, previous coronary heart disease, previous smoker, current smoker, creatinine level, diabetes mellitus, low-density lipoprotein (LDL) cholesterol, the number of transplants, and the total time on kidney replacement therapy (Fig 2). Secondary endpoints were arterial stiffness, traditional CVD risk factors in KTRs (blood pressure, lipid profiles, and eGFR), acute rejections, allograft loss, CV events, and patient survival. Blood samples were drawn at a fasting state in the morning at baseline and at the end of study visits for the measurement of kidney function and CV biomarkers: creatinine, high-sensitivity C-reactive protein, total cholesterol, high-density lipoprotein cholesterol, LDL cholesterol, triglycerides, apolipoprotein (Apo)B, and ApoA1. Arterial stiffness was assessed at the same time points by measuring the central pulse pressure, pulse wave velocity, and augmentation index using the SphygmoCor (ATCOR) method.^30^

### Sample Size and Randomization

We performed a power calculation hypothesizing that the intervention arm decreases the risk of MACE by 30%. We came to that estimate by extrapolation of the reduction in the calculated risk in the previously mentioned study by Soveri et al^27^; the calculated risk of MACE for BENEFIT patients decreased by 31.2% (from 14.3%-10.9%), and that of mortality by 40% (17.5%-12.5%). The corresponding reduction in risk for BENEFIT-EXT patients was 27.8% (22.5%-17.6%) and 22.6% (30.9%-25.2%). For a 2-sample *t* test on a 2-sided significance level of 0.05, assuming a standard deviation of 0.64 (on the natural logarithmic scale), a sample size of 51 per group was required to obtain a power of 0.8 (80%) to detect a 30% calculated risk reduction in MACE. The analysis of covariance (ANCOVA) model was expected to have a slightly greater power than the 2-sample *t* test; therefore, a sample size of 102 patients was seen as sufficient for this study. To account for 8% drop-out, a total of 110 patients, 55 per treatment arm, were included in the study. Randomization to treatment arm was performed using a computerized procedure, stratified by the center, in a 1:1 ratio.

### Statistical Analysis

Owing to a skewed distribution, the primary variable (the estimated risk for MACE and morality) was log transformed (natural logarithm). The primary endpoint was the comparison of the log of the estimated MACE and mortality risk between the treatment groups (CNI- vs belatacept-based immunosuppression) at 1 year. For patients who discontinued the study before 1 year, the last available estimate of CV risk was used in the analysis of the intention-to-treat (ITT) population.

The primary analysis on the primary endpoint was performed using ANCOVA with treatment as a group variable and the baseline log risk for MACE and mortality and center as the covariate. All other comparisons on primary and secondary endpoints were based on ITT comparisons of treatment groups using the 2-sample *t* test or ANCOVA, with correction for baseline variables and/or center. A 2-sided *P* value of <0.05 was considered statistically significant. Analyses were performed using SAS version 9.4 software (SAS Institute).

## Results

### Study Participants and Characteristics

**Figure 3:**
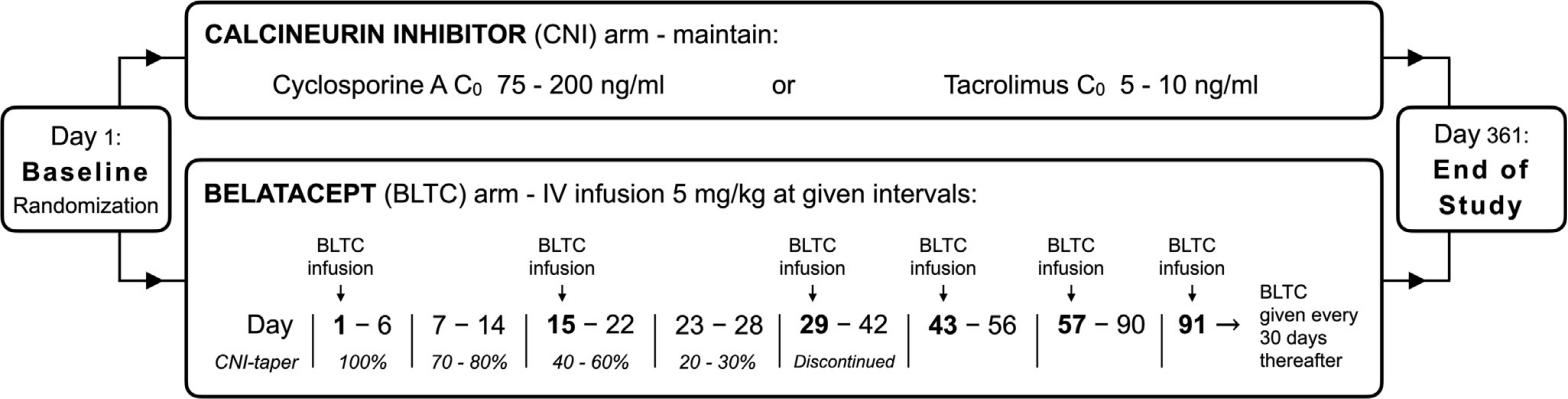
Study flow chart. Abbreviation: IV, intravenous.

A total of 112 patients from 9 centers signed the patient informed consent form. Of these, 1 patient was a screen failure (history of rejection) and was never randomized. Of the 111 randomized patients, 6 withdrew the consent before any study drug was given, 4 in the belatacept arm and 2 in the CNI arm. Thus, 105 patients were administered study medication: 54 in the belatacept arm and 51 in the CNI arm (defining our ITT population). In the belatacept arm, 5 patients were withdrawn from the study: 3 withdrew owing to adverse events (AEs), 1 withdrew consent, and 1 moved out of the country. Similarly, there were 2 withdrawals in the CNI arm; 1 withdrew owing to AEs and 1 withdrew consent. The remaining 49 patients in each treatment arm were defined as the per protocol (PP) population (Fig 3). Because the difference between the PP population and the ITT population was quite small, we did not perform PP analyses to avoid the risk of type I errors caused by multiple comparisons. The first patient was enrolled on September 18, 2014, and the last patient completed the study on September 13, 2018. Baseline demographic data and clinical characteristics for each group are presented in Table 1.

**Table 1:** Baseline Demographics and Clinical Characteristics (ITT population)

	Belatacept Arm (n=54)	CNI Arm (n=51)
Female	13 (24%)	13 (25%)
Age (y)	55.0 (15.2)	54.2 (13.8)
BMI (kg/m^2^)	26.1 (4.1)	27.1 (4.1)
Kidney replacement therapy		
Number of transplantations	1 (1-2)	1 (1-2)
Time since last transplantation (mo)	25.3 (3.7-59.6)	23.4 (3.1-58.8)
Total time on kidney replacement therapy (mo)	35.6 (12.1-489.5)	36.8 (5.3-220.8)
Prior immunosuppressive therapy		
Tacrolimus	53 (98%)	48 (94%)
Cyclosporine	1 (2%)	3 (6%)
Steroids	50 (93%)	50 (98%)
Mycophenolate	50 (93%)	47 (92%)
mTOR inhibitor	3 (6%)	1 (2%)
Baseline immunosuppression trough levels		
Tacrolimus	5.8 (1.7)	5.7 (1.7)
Cyclosporine	94 (4.8)	89 (5.1)
Cardiovascular medication		
ACE inhibitors /angiotensin II antagonists	39 (72%)	29 (57%)
Calcium channel blockers	34 (63%)	39 (77%)
Diuretics	16 (30%)	15 (30%)
α-Adrenoceptor antagonists	11 (20%)	7 (14%)
β-Adrenoceptor antagonists	29 (54%)	27 (53%)
Nitrate	1 (2%)	3 (6%)
Statins	27 (50%)	32 (63%)
Diabetes mellitus	12 (22%)	7 (14%)
Hypertension	30 (56%)	33 (65%)
Systolic blood pressure (mm Hg)	137 (17.2)	133 (18.4)
Diastolic blood pressure (mm Hg)	84 (9.7)	81 (11.2)
Smoking habits		
Nonsmoker	29 (54%)	21 (41%)
Current smoker	6 (11%)	8 (16%)
Previous smoker	19 (35%)	22 (43%)
Cardiovascular disease		
Peripheral vascular disease	8 (15%)	7 (14%)
Previous heart failure	2 (4%)	3 (6%)
Previous coronary heart disease	4 (7%)	6 (12%)
Previous cerebrovascular disease	2 (4%)	4 (8%)
Plasma creatinine (μmol/L)	135 (35.7)	125 (42.6)
eGFR (mL/min/1.73 m^2^)	49.4 (14.8)	56.6 (19.1)
hs-CRP (mg/L)	3.1 (4.1)	2.8 (2.8)
Plasma glucose (mmol/L)	6.2 (1.8)	5.9 (1.7)
Total cholesterol (mmol/L)	5.0 (1.0)	5.1 (1.0)
HDL cholesterol (mmol/L)	1.5 (0.5)	1.6 (0.6)
LDL cholesterol (mmol/L)	2.7 (0.9)	2.7 (0.9)
Triglycerides (mmol/L)	1.9 (0.9)	1.9 (0.9)
Apolipoprotein B (g/L)	1.0 (0.4)	1.0 (0.3)
Apolipoprotein A1 (g/L)	1.4 (0.3)	1.5 (0.4)

*Note:* Data are presented as numbers (percentage) for categorical data, as mean values (standard deviation) for continuous variables, and as medians (min-max) for kidney replacement therapy.

Abbreviations: ACE, angiotensin-converting enzyme; BMI, body mass index; CNI, calcineurin inhibitor; eGFR, estimated glomerular filtration rate; HDL, high-density lipoprotein; hs-CRP, high-sensitivity C-reactive protein; ITT, intention-to-treat; LDL cholesterol, low-density lipoprotein cholesterol; mTOR, mammalian target of rapamycin inhibitor.

### Estimated Risk of MACE and Mortality

The primary endpoint was the estimated 7-year risk of MACE and all-cause mortality per the risk calculator developed by Soveri et al. (Fig 2). After 12 months of treatment, there was no statistically significant difference between the treatment groups in terms of the change in the predicted risk, neither for MACE nor for mortality (Table 2).

**Table 2:** Estimated 7-Year Risk of MACE and Mortality

	Belatacept Arm		CNI Arm		Difference
	Baseline	End of study	Baseline	End of study	
**MACE**					
Mean (SD)	0.15 (0.13)	0.15 (0.15)	0.14 (0.14)	0.15 (0.15)	
Log mean risk change [95% CI]	−2.31 [−2.40, −2.23]		−2.25 [−2.33, −2.16]		0.06 [−0.04, 0.16]
**Mortality**					
Mean (SD)	0.21 (0.19)	0.23 (0.20)	0.19 (0.18)	0.21 (0.19)	
Log mean risk change [95% CI]	−1.94 [−1.96, −1.91]		−1.92 [−1.94, −1.90]		0.02 [−0.01, 0.05]

Abbreviations: CI, confidence interval; CNI, calcineurin inhibitor; MACE, major adverse cardiac event; SD, standard deviation.

In the belatacept arm, the mean (standard deviation) estimated 7-year risk of MACE at baseline was 0.15 (0.13), and it remained unchanged even after 1 year to 0.15 (0.15). Similarly, the risk estimation for the CNI continuation arm was 0.14 (0.14) at baseline and 0.15 (0.15) after 1 year. After applying the ANCOVA models and adjusting for hospital centers, the log mean risk prediction decreased by 2.31 (95% confidence interval [CI]: 2.23, 2.40) for the belatacept group and 2.25 (95% CI: 2.16, 2.33) for the CNI group. The difference between interventions in log mean risk prediction for MACE was 0.06 (95% CI: −0.04, 0.16).

The estimated 7-year mortality risk in the belatacept arm at baseline was 0.21 (0.19), which increased nonsignificantly to 0.23 (0.20) after 1 year. Correspondingly, for the CNI continuation arm, the predicted risk of mortality was 0.19 (0.18) at baseline and increased nonsignificantly to 0.21 (0.19) after 1 year. After applying the ANCOVA models and adjusting for hospital centers, the log mean risk prediction decreased by 1.94 (95% CI: 1.91, 1.96) for the belatacept group and 1.92 (95% CI: 1.90, 1.94) for the CNI group. The difference between interventions in log mean risk prediction for mortality was 0.02 (95% CI: −0.01, 0.05). An overview of the variables used in risk calculation is presented in Table 3.

**Table 3:** Overview of Variables Composing Estimated Cardiovascular Risk

Risk calculator composite	Variable	Belatacept Arm		CNI Arm	
		Baseline	End of study	Baseline	End of study
Common for MACE and mortality	Age (y)	54.5 (15.2)	55.5 (15.2)	53.8 (13.7)	54.8 (13.7)
	Creatinine (μmol/L)	135.1 (35.7)	132.2 (44.1)	124.7 (42.6)	119.1 (38.4)
	Diabetes mellitus	12 (22.2%)	12 (22.2%)	7 (13.7%)	7 (13.7%)
	Coronary HD	4 (7.4%)	4 (7.4%)	6 (11.8%)	6 (11.8%)
	Current smoker	6 (11.1%)	6 (11.1%)	8 (15.7%)	8 (15.7%)
	Previous smoker	19 (35.2%)	19 (35.2%)	22 (43.1%)	22 (43.1%)
MACE only	LDL cholesterol (mmol/L)	2.7 (0.9)	2.6 (1.0)	2.7 (0.9)	2.6 (0.8)
	No. of transplants: 1	51 (94.4%)	51 (94.4%)	48 (94.1%)	48 (94.1%)
	No. of transplants: 2	3 (5.6%)	3 (5.6%)	3 (5.9%)	3 (5.9%)
Mortality only	Total time KRT (mo)	51.4 (69.5)	62.9 (69.6)	45.1 (37.0)	56.9 (37.0)

*Note:* Data are presented as numbers (percentage) for categorical data and as mean values (standard deviation) for continuous variables.

Abbreviations: CNI, calcineurin inhibitor; HD, heart disease; HDL cholesterol, high-density lipoprotein cholesterol; KRT, kidney replacement therapy; MACE, major adverse cardiac event.

Subgroup analysis was also performed to investigate whether the time since transplantation influenced the results in risk calculation. Treatment arms were divided on the basis of the median time after transplantation, thus creating an early and late group (before and after 26 months). There was no difference between the belatacept and the CNI group in the calculated risk of MACE (*P* = 0.33) and mortality (*P* = 0.56) in the subgroups.

### Traditional CVD Risk Factors

The changes in traditional CV biomarkers from the baseline to the end of the study are presented in Table 4. The mean changes were similar between the treatment groups, except for a significant difference in diastolic blood pressure, with lower levels after the belatacept treatment compared with the CNI treatment. Systolic blood pressure showed a similar reduction; however, the difference was not statistically significant.

**Table 4:** Change From Baseline for Traditional CVD Risk Factors

Risk Factor	Belatacept Arm	CNI Arm	Difference	*P* Value
Systolic BP (mm Hg)	−3.3 (−9.18, 2.68)	2.9 (−1.18, 6.97)	−6.2 (−13.26, 0.97)	0.09
Diastolic BP (mm Hg)	−2.6 (−5.38, 0.19)	2.8 (−0.24, 5.88)	−5.4 (−9.50, −1.33)	0.01
hs-CRP (mg/L)	4.64 (−0.94, 10.23)	0.81 (−0.56, 2.18)	3.83 (−1.90, 9.57)	0.19
Plasma glucose (mmol/L)	0.04 (−0.68, 0.77)	−0.06 (−0.65, 0.54)	0.1 (−0.83, 1.03)	0.83
eGFR (mL/min/1.73 m^2^)	1.40 (−0.81, 3.60)	0.73 (−1.50, 2.95)	0.67 (−2.42, 3.76)	0.67
Total cholesterol (mmol/L)	0.22 (−0.55, 0.98)	−0.09 (−0.31, 0.14)	0.3 (−0.49, 1.10)	0.45
HDL cholesterol (mmol/L)	−0.10 (−0.18, −0.03)	−0.02 (−0.09, 0.05)	−0.09 (−0.19, 0.01)	0.08
LDL cholesterol (mmol/L)	−0.10 (−0.31, 0.11)	−0.05 (−0.22, 0.13)	−0.05 (−0.32, 0.22)	0.71
Triglycerides (mmol/L)	−0.06 (−0.29, 0.17)	−0.05 (−0.25, 0.14)	−0.01 (−0.31, 0.29)	0.96
Apolipoprotein B (g/L)	−0.09 (−0.18, −0.00)	−0.06 (−0.13, 0.01)	−0.03 (−0.14, 0.08)	0.59
Apolipoprotein A1 (g/L)	0.02 (−0.07, 0.10)	0.01 (−0.08, 0.09)	0.01 (−0.11, 0.13)	0.88

*Note:* Data are presented as means (95% CI). *P* value results from 2-sample *t* tests.

Abbreviations: BP, blood pressure; CI, confidence interval; CNI, calcineurin inhibitor; CVD, cardiovascular disease; eGFR, estimated glomerular filtration rate; HDL cholesterol, high-density lipoprotein cholesterol; hs-CRP, high-sensitivity C-reactive protein; LDL cholesterol, low-density lipoprotein cholesterol.

### Arterial Stiffness

Arterial stiffness was measured at baseline and at the end of the study using the SphygmoCor method. Compared with the CNI group, the central diastolic pressure in patients of the belatacept group decreased by 6.55 mm Hg (95% CI: 1.83, 11.27; *P* = 0.01) after 1 year of treatment. For central systolic pressures, the difference of 6.1 mm Hg between the study groups (95% CI: −0.11, 12.34; *P* = 0.05) was borderline significant. There were no differences between the treatment arms in the central pulse pressure, pulse wave velocity, and augmentation index (Table 5).

**Table 5:** Change From Baseline in Arterial Stiffness Variables

Risk Factor	Belatacept Arm	CNI Arm	Difference	*P* Value
Augmentation index (%)	−1.26 (−4.51, 1.99)	1.04 (−2.41, 4.48)	−2.30 (−6.96, 2.37)	0.33
Pulse wave velocity (cm/s)	−0.44 (−1.02, 0.13)	0.1 (−0.88, 1.08)	−0.54 (−1.67, 0.58)	0.34
Central systolic pressure (mm Hg)	−4.45 (−9.09, 0.18)	1.65 (−2.63, 5.94)	−6.1 (−12.33, 0.11)	0.05
Central diastolic pressure (mm Hg)	−3.72 (−7.41, −0.02)	2.83 (−0.17, 5.84)	−6.55 (−11.24, −1.86)	0.01
Central pulse pressure (mm Hg)	−0.60 (−4.03, 2.83)	−0.37 (−3.51, 2.77)	−0.23 (−4.81, 4.35)	0.92

*Note:* Data are presented as means (95% CI). *P* value results from 2-sample *t* tests.

Abbreviations: CI, confidence interval; CNI, calcineurin inhibitor.

### CV Events and Patient Survival

During the 1-year study period, there were no CV events (including CV death, nonfatal myocardial infarction, nonfatal stroke, hospitalization because of congestive heart failure or angina pectoris, or coronary intervention) or deaths observed in the study population.

### Safety Evaluation

All patients in both study groups reported at least 1 AE during the duration of the study (Table 6). The majority of the events were of mild severity and considered unrelated to the study drug. More patients in the belatacept group (53.7% vs 21.6%) reported AEs that were considered possibly or probably related to the intervention. Three patients in the belatacept group and 1 patient in the CNI continuation group reported AEs that led to their withdrawal from the study. Serious AEs were reported by 29.6% of the patients in the belatacept group compared with 15.7% in the CNI group. Patients allocated to the belatacept group had more infections (Table 7). There was 1 case of incident cancer (lung cancer), which occurred in the belatacept group.

**Table 6:** Number and Proportion of Patients With Adverse Events

	Belatacept Arm		CNI Arm	
	n	%	n	%
Any adverse event	54	100	51	100
1 adverse event	10	18.5	21	41.2
>1 adverse events	44	81.5	30	58.8
Any possible or probable intervention-related adverse events	29	53.7	11	21.6
Adverse events leading to withdrawal	3	5.6	1	2.0
Serious adverse events	16	29.6	8	15.7
Suspected acute rejection	7	13.0	1	2.0
Biopsy-proven acute rejection	4	7.4	1	2.0
Graft loss due to acute rejection	1	1.9	0	-
Cancer	1	1.9	0	-

Abbreviation: CNI, calcineurin inhibitor.

**Table 7:** Adverse Events Reported by ≥5% of Patients in Either Treatment Group

Event	Belatacept Arm (n=54)	CNI Arm (n=51)
Urinary tract infection	19 (35%)	4 (8%)
Pyrexia	17 (32%)	1 (2%)
Abdominal pain/discomfort	10 (19%)	1 (2%)
Nasopharyngitis	10 (19%)	8 (16%)
Respiratory tract infection	8 (15%)	5 (10%)
Coughing	8 (15%)	1 (2%)
Edema	5 (9%)	3 (6%)
Diarrhea	5 (9%)	2 (4%)
Anemia	5 (9%)	1 (2%)
Fatigue	4 (7%)	1 (2%)
Headache	4 (7%)	1 (2%)
Dizziness	4 (7%)	0
Arthralgia	2 (4%)	3 (6%)
Gastroenteritis	1 (2%)	3 (6%)
Nausea	3 (6%)	1 (2%)
Herpes zoster	3 (6%)	1 (2%)
Myalgia	3 (6%)	1 (2%)
Aphthous ulcer	3 (6%)	0

*Note:* Incidence rates given in numbers (percentage).

Abbreviation: CNI, calcineurin inhibitor**.**

During the study, 8 acute rejection episodes were suspected, and graft biopsies were obtained for further investigation. Acute rejection was confirmed in 4 of the 7 suspected cases in the belatacept group, and in a single case in the CNI group. Three of the rejection episodes were considered severe (Banff grade IIA or higher): 2 in the belatacept group and 1 in the CNI-treated group. One patient (belatacept) proved refractory despite the antirejection treatment with methylprednisolone and T-cell–depleting antibodies. All other rejection episodes recovered on treatment with corticosteroids or antithymocyte globulin as per local practices.

## Discussion

In this randomized study, in which stable kidney transplant patients were converted from a CNI-based maintenance immunosuppressive regimen to belatacept, no difference in the calculated 7-year risk of MACE or all-cause mortality could be demonstrated even after 1 year of follow-up. We were unable to find a significant effect on any of the 3 modifiable CV risk factors that were used as input variables in the risk calculator (serum LDL cholesterol, diabetes prevalence, and serum creatinine level). The belatacept arm had significantly lower diastolic blood pressure, measured both centrally (SphygmoCor method) and peripherally. We found a similar improvement in systolic pressure (Table 4); however, this difference was not statistically significant (*P* = 0.09).

Of the 3 modifiable risk factors in the calculator, we had expected a significant change in eGFR in the intervention arm. Our findings are in contrast with those of the BENEFIT studies, as well as other belatacept-conversion studies reported in the literature.^24^^,^^31^, ^32^ In those studies, there was a consistent improvement in graft function on conversion to belatacept. One possible explanation for this was the predominant use of TAC by our study participants, with relatively low trough levels (Table 1) at baseline. In the Symphony trial,^33^ the low-dose TAC group had an average trough level of 6.7 ng/mL 1 year after transplantation and achieved an eGFR on average 5.7 mL/min/1.73 m^2^ higher than the other 3 comparator groups. A belatacept-conversion study by Grinyo et al^34^ examined 173 patients with a mean time after transplantation to randomization of 19 months, a baseline eGFR of 54 mL/min/1.73 m^2^, and a low immunologic risk profile, making the population reasonably comparable to ours. Belatacept patients in that study showed an average improvement in eGFR of 4.9 mL/min/1.73 m^2^ compared with CNI patients. At baseline, patients using TAC (56%) had an average trough level of 7.2 ng/mL, whereas patients on CsA (44%) had an average trough level of 160.2 ng/mL. In our study, the mean trough levels of TAC (5.7 ng/mL) and CsA (91 ng/mL, 4 patients only) at the time of randomization were lower compared with those in both these studies.^33^^,^^34^ The lower CNI trough levels may have already significantly decreased the nephrotoxic side effects and explain why our belatacept patients only experienced a nonsignificant gain in eGFR of 0.7 mL/min/1.73 m^2^. However, this hypothesis is not supported by Budde et al^35^ in a recent study that used a very similar design to ours. Their results showed an average TAC trough level of 6.27 mg/L and 5.82 mg/L, respectively, at baseline and after 1 year in the control arm. The average difference in eGFR between belatacept and TAC groups at that point was already 6.8 mL/min/1.73 m^2^, contrary to our findings. Of note, patients in our study with biopsy-proven acute rejection (BPAR) had a decrease in eGFR of 2.7 mL/min/1.73 m^2^, compared with an increase of 1.1 mL/min/1.73 m^2^ in others. The low number of events precludes any meaningful interpretation of these data, and the general lack of improvement in eGFR cannot be explained hereby.

The second element of the calculator is diabetes status. Multiple studies have corroborated the diabetogenicity of TAC in transplantation.^36^, ^37^, ^38^ Furthermore, reversibility of beta-cell dysfunction and posttransplant diabetes mellitus after TAC withdrawal has been established in both animal studies and clinical experience.^39^, ^40^, ^41^, ^42^ Thus, we expected an improvement in glycemic metabolism on conversion from TAC to belatacept. However, no participant in our study reversed diabetes mellitus or developed posttransplant diabetes mellitus in either study arm (Table 3). Also, triglyceride, serum ApoB, and serum ApoA1 levels did not improve (Table 4), which is of interest, because all 3 of these parameters are mentioned as risk factors for developing posttransplant diabetes mellitus.^43^^,^^44^

Expectations regarding the effect on lipid profile, the third element in the calculator, were limited. Although CsA has been implicated in dyslipidemia,^45^ TAC seems to be less detrimental to lipid status. In our study, 94% of the participants were on TAC before randomization. Ferguson et al^46^ compared 3 steroid-avoiding regimens of immunosuppression: belatacept with MMF versus belatacept with sirolimus versus TAC with MMF. Both the belatacept arms had a lower LDL level (23.9 and 25.0 mg/mL vs 34.0 mg/mL for TAC with MMF) after 1 year; however, the difference was nonsignificant, possibly related to the limited sample size of the study. Another observational study focusing on the metabolic effects of conversion from TAC to belatacept found improvement in the GFR and acid-base status, but not in blood lipids.^47^ Our findings are in line with these reports, because we found no effect on the LDL cholesterol level (Table 3).

On trying to explain the lack of significant improvement in CV risk, we need to consider another bias besides the low CNI trough levels. Patients were already treated with CNI for a median of 26 months since transplantation. Serious negative side effects of CNI treatment could be less likely found in the control group, because patients suffering from these side effects could have been converted to alternative immunosuppression earlier on and thus not be eligible for this study. The only positive effect that we found for belatacept was a significant improvement in diastolic blood pressure, measured both centrally (SphygmoCor method) and peripherally. For systolic pressure, a similar improvement was found (Table 4); however, it was not statistically significant (*P* = 0.09), most likely owing to the relatively small sample size of this study. CNIs are known as potent vasoconstrictors and contribute to posttransplant hypertension.^48^ A previous study comparing the effects of belatacept and CsA on central aortic blood pressure and arterial stiffness after kidney transplantation has shown no differences in blood pressure, both centrally and peripherally, in the 2 groups.^49^ However, the study was also hampered by a low sample size and may have lacked the power to detect significant differences. Although not included in the calculator, blood pressure is of course an established risk factor for CVD. Moreover, high blood pressure is strongly associated with the risk of graft failure, and finding an improvement in this parameter could still indicate an advantage for belatacept treatment.^50^

Regarding safety, AEs occurred in both the groups; however, serious AEs were reported almost twice as often in belatacept-treated patients (29.6% vs 15.7%), and they were more likely to discontinue their study treatment than patients treated with CNI (5.6% vs 2.0%). Rejection was seen more often in the belatacept patients. Four episodes of BPAR occurred in the belatacept group versus 1 single episode in the CNI group (7.4% vs 2.0%). Three patients showed signs of vascular inflammation in the biopsy, corresponding to Banff grade II, 2 of whom were in the belatacept group. All 3 patients were treated according to the local protocol with high-dose steroids and T-cell–depleting antibodies, despite which 1 belatacept patient suffered graft loss and reinitiated the dialysis treatment. The other 2 patients recovered with antirejection treatment. The median number of days between transplantation and randomization was 225 in those treated with belatacept with BPAR, whereas no rejection was seen in any patient after 1 year after transplantation (mean 818 days after transplantation for those without BPAR). This trend of rejection after early conversion corroborates the findings by Budde et al,^35^ in which all belatacept rejections were seen within 1 year of transplantation.

The rate of rejection in this study is in line with that in earlier reports. For example, in the trial by Grinyo et al,^34^ 7.1% of belatacept patients experienced rejection versus none in the CNI group. In another trial by Adams et al,^51^ 1-year rejection rates were around 50% when belatacept was used right after transplantation, declining to 33% when TAC was tapered off 3-5 months after transplantation. When TAC was tapered after 11 months, the rejection rates between TAC- and belatacept-treated patients were similar, around 16%. Other reports have described varying (0%-11%) rates of rejection; however, these are data from nonrandomized “rescue” settings after even longer time after transplantation and are therefore comparable with our results.^52^^,^^53^

Besides rejection, urinary tract infections, nasopharyngitis, and other respiratory tract infections were more often seen in the belatacept arm (Table 7). The present study’s planned visits could have led to a bias in the reporting of uncomplicated infections, because a study visit was planned every month for belatacept patients, instead of every 3 months for the CNI continuation group.

Not a single case of pneumocystis-jirovecii pneumonia, cytomegalovirus, polyoma- or Epstein-Barr virus–associated disease was seen in the belatacept patients. Three cases of cytomegalovirus infection were seen in CNI patients. Previous reports have been inconclusive on opportunistic infections in belatacept treatment. The follow-up study to the first belatacept-conversion trial noted a slightly higher incidence of viral infection (11% vs 14%).^34^ In a recent study by Bertrand et al,^54^ 50 opportunistic infections were noted in 453 patients treated with belatacept (9.8%). In a multivariate analysis of that study, the authors concluded that patients with a low GFR (<25 mL/min/1.73 m^2^) and patients converted early after transplantation (within 6 months) were more likely to develop opportunistic infections.

There was one case of lung cancer in the belatacept group in the present study. Previous studies have not indicated a higher risk of malignancy for belatacept beyond posttransplant lymphoproliferative disorders.^24^^,^^34^

A major strength of the current study is the international multicenter approach, making it representative of the European transplantation practice. However, this study also has important limitations, which must be considered. The study duration of 1 year was most likely too short to show a significant difference in kidney function between the 2 study groups. We have overestimated the potential reduction in MACE and mortality in patients who use low-dose TAC instead of CsA. Another limitation was the heterogeneous time from transplantation to trial enrollment, and the small number of patients treated with CsA and the relatively large span of eGFR at baseline also contributed to the heterogeneity. Patients with a severely diminished graft function were less likely to benefit from conversion.

In conclusion, we have shown no effect on the calculated CV risk or kidney function in this study comparing conversion to belatacept with continuation of CNI-based immunosuppression. We did show a significant difference in diastolic blood pressure. We reconfirmed the increased chance of rejection when converting to belatacept. After more than 10 years of clinical experience, the place of belatacept in kidney transplantation is still not fully established; however, it may be an attractive option when patients suffer from significant side effects of CNI, such as nephrotoxicity or posttransplant diabetes mellitus. However, it is hard to define a significant benefit of belatacept for patients who do well on a low-dose TAC-based therapy without severe CNI-related side effects. Further studies are needed to define the place of belatacept in kidney transplantation.
